# *In Vitro* Effect of Fenugreek Extracts on Intestinal Sodium-dependent Glucose Uptake and Hepatic Glycogen Phosphorylase A

**DOI:** 10.1155/EDR.2001.91

**Published:** 2001

**Authors:** M. Al-Habori, A. Raman, M. J. Lawrence, P. Skett

**Affiliations:** 1 Department of Clinical Biochemistry University of Sana'a Sana'a Yemen; 2 Department of Pharmacy King's College London Franklin-Wilkins Building 150 Stamford Street London SE1 8WA UK; 3 Institute of Biomedical and Life Sciences West Medical Building University of Glasgow Glasgow G12 8QQ UK

## Abstract

Fenugreek (*Trigonella foenum-graecum* L. seed) is a food with traditional medicinal use in diabetes. Beneficial effects have been demonstrated in diabetic animals and both insulin-dependent and non-insulin-dependent diabetic subjects. Effects of a lipid extract A, crude ethanolic extract B, further sub-fractions of B (saponin-free C, saponin D and sapogenin E) and a gum fibre fraction F on intestinal sodium-dependent glucose uptake were investigated *in vitro* using rabbit intestinal brush border membrane vesicles. All fractions except A inhibited glucose-uptake at 0.33 and/or 3.3mg/mL (*p* < 0.001). Greatest inhibition was observed with fractions D and E. Diosgenin and trigonelline (compounds reported in fenugreek)also inhibited glucose-uptake (^50^C values approximately 3mg/ ml, equivalent to 8mM and 19mM respectively) but did not account for the activity of the crude extracts. Fenugreek extracts had no effect on basal levels of glycogen phosphorylase a (HGPa) activity in rat hepatocyte suspensions. However fractions C and E caused a marginal but statistically significant inhibition (18.9 and 15.1% respectively, *p* < 0.05) of glucagon induction of this enzyme suggesting a glucagon-antagonist effect. Diosgenin (1.65mg/ml; 4mM) inhibited glucagon-induced HGPa activity by 20% (*p* < 0.05), and was more effective than trigonelline (non significant inhibition of 9.4% at 1.65mg/ml, 10mM).


Int. Jnl. Experimental Diab. Res., Vol. 2, pp. 91–99
Reprints available directly from the publisher
Photocopying permitted by license only

© 2001 OPA (Overseas Publishers Association) N.V.
Published by license under
the Harwood Academic Publishers imprint,
part of Gordon and Breach Publishing,
a member of the Taylor & Francis Group.
Printed in the U.S.A.

### *In Vitro* Effect of Fenugreek Extracts on Intestinal Sodium-dependent Glucose Uptake and Hepatic Glycogen Phosphorylase A

M. AL-HABORI[a], A. RAMAN[b,*], M. J. LAWRENCE[b] and P. SKETT[c]

[a]*Department of Clinical Biochemistry, University of Sana'a, Sana'a, Republic of Yemen;* [b]*Department of Pharmacy, King's College London, Franklin-Wilkins Building, 150 Stamford Street, London SE1 8WA, UK;*
[c]*Institute of Biomedical and Life Sciences, West Medical Building, University of Glasgow, Glasgow G12 8QQ, UK*

*(Received 9 March 2001; In final form 25 April 2001)*

**Fenugreek (*Trigonella foenum-graecum* L. seed) is a food with traditional medicinal use in diabetes. Beneficial effects have been demonstrated in diabetic animals and both insulin-dependent and non-insulin-dependent diabetic subjects. Effects of a lipid extract A, crude ethanolic extract B, further sub-fractions of B (saponin-free C, saponin D and sapogenin E) and a gum fibre fraction F on intestinal sodium-dependent glucose uptake were investigated *in vitro* using rabbit intestinal brush border membrane vesicles. All fractions except A inhibited glucose-uptake at 0.33 and/or 3.3 mg/mL ($p < 0.001$). Greatest inhibition was observed with fractions D and E. Diosgenin and trigonelline (compounds reported in fenugreek) also inhibited glucose-uptake ($IC_{50}$ values approximately 3 mg/ml, equivalent to 8 mM and 19 mM respectively) but did not account for the activity of the crude extracts. Fenugreek extracts had no effect on basal levels of glycogen phosphorylase a (HGPa) activity in rat hepatocyte suspensions. However fractions C and E caused a marginal but statistically significant inhibition (18.9 and 15.1% respectively, $p < 0.05$) of glucagon induction of this enzyme suggesting a glucagon-antagonist effect. Diosgenin (1.65 mg/ml; 4 mM) inhibited glucagon-induced HGPa activity by 20% ($p < 0.05$), and was more effective than trigonelline (non significant inhibition of 9.4% at 1.65 mg/ml, 10 mM).**

*Keywords*: Fenugreek; *Trigonella*; SGLT-1; BBMV; Glycogen phosphorylase a; Hepatocytes

### INTRODUCTION

Diabetes mellitus affects 3–5% of people living in Western countries and is a heterogeneous disorder with varying prevalence among different ethnic groups.[1] Based on the emerging relationship between hyperglycaemia and degree of glycaemic control on one side and the development of microvascular and macrovascular complications on the other,[2,3] there is a growing interest in the inclusion of beneficial food substances in the diet to assist the control of glucose levels.

*Corresponding author. Tel.: + 44-20-7848-4810, Fax: + 44-20-7848-4800, e-mail: amala.raman@kcl.ac.uk

Fenugreek (*Trigonella foenum-graecum* L.) is an annual plant from the family Papilionaceae-Leguminosae, and is extensively cultivated as a food crop in India, the Mediterranean region, North Africa and Yemen. Main uses of the seeds (often referred to as "fenugreek") are as a spice and, when defatted, as a flour. Fenugreek has also been credited with many medicinal properties (reviewed by AL-Habori and Raman, 1998).[4] In particular, anti-diabetic and hypocholesterolaemic effects have been demonstrated in diabetic animals and both insulin-dependent and non-insulin-dependent (Type I and Type II) diabetes mellitus.[4,5,6,7] For example,[6] consumption of 15g of ground fenugreek 15 minutes before a standardised meal, reduced the postprandial elevation of plasma glucose by an average of 20% ($p < 0.05$) in 17 out of 21 Type II patients. In a group of 11 Type I patients, consumption of 100 g of fenugreek daily for 10 days resulted in a lowering of fasting blood glucose from 15.1 to 10.9 mM, a 20% decrease in the area under the glucose tolerance curve and a 54% fall in 24-hour urinary sugar excretion.[7]

The anti-diabetic activity of fenugreek is primarily associated with the defatted fraction of the seeds,[8,9,10] and can be largely attributed to their saponin and high fibre content but not to the major alkaloid trigonelline.[5,6,11] Suggested mechanisms for fenugreek's anti-diabetic effects include delayed gastric emptying and interference with glucose absorption due to high fibre content,[5,6,11] inhibition of pancreatic $\alpha$-amylase and intestinal disaccharidase enzymes,[12,13] or the presence of an orally active principle capable of inducing hypoglycaemia[14,15] or enhanced plasma insulin levels.[16,17] The compound 4-hydroxyisoleucine, which represents up to 80% of free amino acids in fenugreek seeds, was found to possess insulin-secretagogue properties both in pancreatic islets *in vitro* and in rats and dogs *in vivo*.[18,19]

In the light of the reported beneficial role of fenugreek in the management of diabetes, and its complex chemical profile, the aim of this study was to investigate further the possible mechanisms and target sites through which fenugreek may act, by making use of *in vitro* models. We have prepared a range of fractions to investigate the role of different fenugreek constituents in its anti-diabetic activity. Most studies[4] have employed the defatted seeds. However, we have also investigated the activity of the lipid fraction which would normally be discarded. The defatted ethanol extract (used in many studies) would be expected to contain saponins and other polar materials. We have used n-butanol to separate the saponins from water-soluble constituents of fenugreek. Since saponins are likely to undergo hydrolysis in the gastrointestinal tract an aglycone (sapogenin) extract was prepared. Finally we have also tested the effect of trigonelline and diosgenin (a sapogenin) two well known compounds derived from fenugreek.

The effects of the fenugreek fractions on intestinal glucose uptake was investigated using isolated intestinal brush border membrane vesicles (BBMVs) prepared from the apical membrane microvilli of rabbit intestinal enterocytes[20,21] and effects on liver glycogen degradation examined using isolated rat hepatocyte suspensions. A polysaccharide gum fibre fraction was tested in the intestinal model alone since intact polysaccharides would not be expected to reach the liver.

There is evidence from rat models that the expression of the sodium-dependent glucose transporter (SGLT-1) and a sodium-independent transporter GLUT-2 is raised in the diabetic state, thus increasing the likelihood of hyperglycaemia following a meal.[22,23,24] The major component of glucose transport in rat isolated enterocytes was found to be due to SGLT-1[25] which can be blocked by the plant-derived glycoside phloridzin (phlorizin).[26] BBMV comprise spherical vesicles in which SGLT-1 is expressed in the membrane, the outer surface representing the intestinal lumen and the core of the vesicle the intestinal cell. The advantage of using BBMV

in such studies is that SGLT-1-mediated glucose transport can be studied uncomplicated by interference from GLUT-2 transporters[27] or cytoplasmic glucose metabolism.

Isolated hepatocyte suspensions provide a good model for studying insulin-like or glucagon-antagonistic activity. One enzyme that may be studied is hepatic glycogen phosphorylase a (HGPa), which causes the degradation of glycogen to glucose-1-phosphate. However, *in vitro*, the activity of the enzyme is studied using the reverse reaction. Insulin has been shown to reduce the activity of this enzyme in the absence of glucagon as well as counteracting the glucagon stimulation of HGPa.[28]

### MATERIALS AND METHODS

### Materials

All chemicals were obtained from Sigma Chemical Company (Poole, Dorset, UK). Commercial fenugreek seed was purchased from an Indian supermarket in North London. A voucher specimen (number Tr 91 Kg) has been deposited in the Pharmacognosy Research Laboratories, Pharmacy Department, Kings College London.

### Extraction and Phytochemical Work

#### *Preparation of Extracts*

Fenugreek seed (1 kg) was ground in a Maig grinder to pass through a 0.8 mm mesh sieve and continuously extracted (Soxhlet apparatus) with light petroleum (40/60°) for 16 hours. The extract was dried by rotary evaporation under vacuum to yield an oil fraction (A; 45 g). The defatted fenugreek seed powder (895 g) was dried at room temperature and continuously extracted with absolute ethanol for 24 hours. Drying as before yielded the crude ethanolic extract (B; 65 g). A portion of B was re-dissolved in water and further extracted by n-butanol (500 ml) in a separating funnel. A saponin extract (C; 16.9 g) was obtained by evaporating the n-butanol layer, leaving the water layer containing saponin-free extract (D; 13.7 g on freeze drying). An aglycone extract (E; 6.75 g) was obtained by hydrolysis of the saponin extract (C; 9.2 g) by refluxing it with $H_2SO_4$ (10%, 20 ml) for 30 minutes and then extracting it with equal volume of chloroform, which was later dried under vacuum.

Gum fibre was extracted according to a previously reported method.[5] Defatted seed powder (1 g) was extracted with acetic acid (5% aqueous, 100 ml) by continual stirring at room temperature for 30 minutes. The mixture was centrifuged (30 minutes; 20,000 rpm) and the supernatant was recovered. The above acetic acid extraction of the sediment and centrifugation was repeated 3-times. Supernatants were pooled together and gum fibre precipitated by addition of absolute ethanol to 50% v/v with continuous stirring. The suspension was centrifuged (10,000 rpm; 15 minutes). The supernatant was decanted and the precipitate washed with absolute ethanol and acetone. The hair-like precipitate was collected by filtration (F; 90 mg).

#### *Preparation of Fractions for Bioassay*

Due to their poor water-solubility, the fractions were solublized in 0.3% v/v Tween-80 for testing. The latter solution was used as the control in these experiments. The fibre fraction produced a thick gel on dissolving in 0.3% Tween 80 and was only tested at a concentration of 0.33 mg/ml.

#### *Flash Chromatography*

The aglycone fraction (E; 1.09 g) was subjected to silica gel flash chromatography (Kieselgel 60, 40–63 μm; 40 g), eluting successively with 200 ml volumes of petroleum ether, petroleum ether-chloroform (1:1), chloroform, chloroform-methanol (19:1, 9:1, 5:1, 4:1, and 1:1) and methanol. Eluate was collected as 100 ml fractions, which were concentrated and compared with parent extract (E) using silica-gel thin-layer chromatography (TLC; solvent: chloroform: methanol, 19:1, plate dried and transferred to a second tank containing chloroform: methanol: water, 65:35:10). TLC zones were detected using anisaldehyde spray reagent (anisaldehyde: methanol: sulphuric acid: acetic acid, 0.5: 85:10:5) followed by heating at 105°C. Fractions of similar chemical constituents were pooled together. The major fraction (G; 0.83 g) eluted with chloroform.

### *Glucose Content of Extracts*

The presence of sugars (glucose, galactose and fructose) in the extracts was determined qualitatively by TLC and quantitatively by high performance liquid chromatography (HPLC).

TLC system: Silica gel G, developed with ethylacetate/acetic acid/methanol/$H_2O$ (60:15:15:10); detection with anisaldehyde spray reagent as described above.

HPLC system: Apex $NH_2$ silica column (5 μ; 15 cm), acetonitrile/$H_2O$ (80:20) mobile phase; refractive index detection; internal standard maltose.

### Intestinal Glucose Uptake Experiments

### *Preparation of Rabbit Intestine*

The small intestine of a New-Zealand White rabbit (killed by administration of sodium pentobarbitone) was dissected from the pyloric sphincter to the ileo-caecal junction and put into ice-cold KCl solution (154 mM). The remaining mesentery and fat were thoroughly removed, the intestine transferred to fresh ice-cold KCl solution and the contents gently squeezed out. The small intestine was cut longitudinally and washed thoroughly in KCl solution. It was then blotted dry with paper tissue, snap frozen in a plastic bag in liquid nitrogen, and stored at −70°C.

### *Preparation of BBMV*

BBMV were prepared from the tips of the microvilli of rabbit cleaned small intestine using the divalent-cation precipitation method[20] as modified by Kessler *et al.* (1978).[21] Frozen small intestine was thawed in Buffer 1 (200 ml; D-mannitol 10 mM, HEPES 2 mM, Tris to pH 7.4). Epithelium was separated from the underlying mucosa by mechanical vibration on a VIBRO mixer at maximum speed (1 min), cooling in ice (1 min), followed by further vibration (30 s). The solution was filtered under vacuum through a Buchner funnel, without filter paper, to remove muscle debris. The filtrate was made up to volume (300 ml) with Buffer 1 and solid $MgCl_2$ (0.61 g) added. After stirring (0°C, 20 min), the suspension was centrifuged (3015 g, 4°C, 15 min). The resultant supernatant was centrifuged (34858 g, 4°C, 30 min) and the supernatant discarded. The pellet was resuspended in Buffer 2 (8 ml; D-mannitol 100 mM, $MgSO_4$ 0.1 mM, HEPES 2 mM, Tris to pH 7.4). The resuspended pellet was centrifuged (34858 g, 4°C, 40 min) and the supernatant discarded. The resultant washed pellet was resuspended in 3 times the pelleted volume in Buffer 3 (mannitol 300 mM, $MgSO_4$ 0.1 mM, HEPES 10mM, Tris to pH 7.4). The suspension was passed 5 times through a 25 gauge needle, aliquoted in 0.26 ml volumes and snap frozen in liquid nitrogen. Prior to use, each aliquot (0.26 ml) of BBMV was diluted with Buffer 3 (0.44 ml). This final suspension will be referred to as the BBMV suspension.

### *BBMV Protein Content*

Protein content of the BBMV suspension (diluted 1:10, 1:50 and 1:100 in water) was assayed against bovine serum albumin using Coomassie blue as per the Bradford method.[29]

### *Glucose Uptake Studies*

Glucose uptake was determined by the rapid-filtration method of Hopfer *et al.*[30] BBMV

suspension (20 μl) was incubated at 25°C with radio-labelled D-glucose in buffer (20 μl; glucose 0.2 mM, NaSCN 200 mM, D-mannitol 200 mM, HEPES 20 mM, Tris to pH 7.4, containing $^3$H-D-glucose 0.3 MBq per ml) and either test (extract) dissolved in 0.3% Tween 80, 0.3% Tween 80 solution, or water (20 μl). Reaction was initiated by addition of BBMV suspension, and terminated at the required time by addition of ice-cold stop-wash buffer (1 ml; NaCl 200 mM, phlorizin 250 μM, 10 mM HEPES, Tris to pH 7.4). The solution was filtered under vacuum through a pre-wetted cellulose acetate/nitrate filter (0.45 μm; Millipore). The sample tube was rinsed with stop-wash buffer (2 × 1 ml) which was also passed through the filter. The filter paper was transferred to a scintillation vial containing Ready-Protein scintillant (Beckman, Ireland). Radioactivity was counted for 300 s in a scintillation counter (LKB Wallac 1209 Rackbeta). The time course of glucose uptake was determined by terminating incubations containing BBMV, glucose buffer and water at short intervals over a one minute period (Fig. 1). Some incubations were continued to 5 minutes or 1 hour to ensure that equilibrium was obtained (Fig. 1). Performance of vesicles was routinely checked by ensuring that a satisfactory peak to equilibrium ratio was obtained.

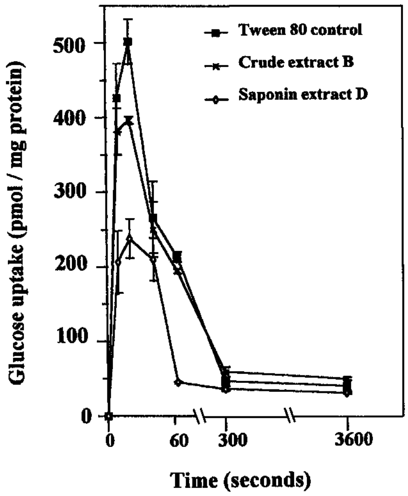

FIGURE 1 Typical glucose uptake profile into rabbit intestinal brush border membrane vesicles (BBMV) in the absence and presence of fenugreek extracts. Each point represents the mean of 6 replicate incubations ± SEM for three separate batches of BBMV.

For calculation of % inhibition, data from incubations terminated at 20 seconds (corresponding to peak glucose uptake) were used. Data were statistically analysed by one way ANOVA and t-test. Glucose uptake was expressed (Tab. I) as pmol glucose/mg protein ± SEM of three different intestinal preparations (n = 6 for each preparation).

TABLE I Glucose uptake into intestinal BBMVs in the presence of extracts of Fenugreek seeds

| | Glucose uptake with test substance at: | | | |
|---|---|---|---|---|
| | 3.3 mg/ml | | 0.33 mg/ml | |
| Test substance | (a) | (b) | (a) | (b) |
| A - Oil fraction | 442.5 ± 17.6 | (88%) | ND | ND |
| B - Crude extract | 362.8 ± 6.9* | (72%) | 469.4 ± 8.0* | (94%) |
| C - Saponin-free extract | 183.2 ± 8.9* | (37%) | 376.7 ± 4.8* | (75%) |
| D - Saponin extract | 116.5 ± 7.1* | (23%) | 392.9 ± 2.6* | (78%) |
| E - Aglycone extract | 94.1 ± 1.4* | (19%) | 348.8 ± 12.2* | (70%) |
| F - Fibre | ND | ND | 307.1 ± 6.3* | (61%) |
| G - Main aglycone fraction | 270.9 ± 7.8* | (54%) | 376.3 ± 11.0* | (75%) |
| Diosgenin | 274.8 ± 10.1* | (55%) | 449.5 ± 18.1 | (90%) |
| Trigonelline | 226.8 ± 10.5* | (45%) | 307.1 ± 8.4* | (61%) |

Data given as mean ± SEM of three separate BBMV preparations (n = 6 for each preparation). Control values 574.7 ± 15.6 and 501.7 ± 14.0§ pmol glucose/mg protein for water and 0.3% Tween 80 respectively. (a) Expressed as pmol glucose/mg protein; (b) expressed as a % of the 0.3% Tween 80 control; ND - not determined. * = $p < 0.001$, with respect to Tween-80 (control); § - not significantly different from water control.

### Isolated Hepatocyte Suspension Experiments

### *Preparation of Hepatocyte Suspensions*

Rat isolated hepatocyte suspensions were prepared by a collagenase perfusion method[31] and adjusted to $3–5 \times 10^6$ cells per ml with Krebs' medium. Aliquots were gassed with $O_2/CO_2$ (19:1) for 15 seconds.

### *Test Incubations*

Following a pre-incubation period of 30 minutes, hepatocyte suspension (450 μl) was transferred to 1 ml microcentrifuge tubes containing either 50 μl of test sample, 50 μl vehicle control, or 25 μl of glucagon (10 μM) and 25 μl of either test sample or vehicle control. Once mixed the sample tubes were incubated at 37°C on a dry heating block for 4 minutes. The incubation was stopped by rapidly freezing the tubes and storing them at −80°C.

### *HGPa Assay*

Crude HGPa was released from hepatocytes by adding an equal volume (500 μl) of ice-cold MOPS buffer (100 mM MOPS, 200 mM NaF, 30 mM NaEDTA, 10 mM dithiothreitiol) to the frozen sample which was then left to thaw on ice. Hepatocytes were sonicated for 60 seconds and the lysate vortexed and centrifuged (10 min; 2500 g; 4°C).

To measure HGPa activity, 50 μl aliquots of the supernatant were transferred to 1 ml microcentrifuge tubes containing 50 μl of radioactive assay mixture (2% glycogen, 1 mM caffeine, 100 mM glucose-1-phosphate, 50 nM $^{14}$C-glucose-1-phosphate (specific activity 50 GBq/mmole). This mixture was vortexed and incubated in a water bath for 20 minutes at 37°C and the reaction stopped by placing the sample tubes on an ice-bath. Aliquots of reaction mixture (30 μl) were spotted onto 1 cm$^2$ filter paper pieces, washed 3 times with 66% (v/v) ethanol in water, followed by a wash in acetone for 1 minute and allowed to dry. The filter paper pieces were transferred into scintillation vials, 5 mL of scintillant fluid (Ecoscintillant A) added and counted for radioactivity. The results were statistically analysed by one way ANOVA and t-test. HGPa activity (Tab. II) is expressed as nmol of $^{14}$C-glucose-1-phosphate incorporated into glycogen per mg of protein per minute ± SEM of three different hepatocyte preparations (n = 3 for each preparation).

TABLE II Effects of Fenugreek seeds extracts on hepatic glycogen phosphorylase a activity

| | Hepatic glycogen phosphorylase a activity | | | |
|---|---|---|---|---|
| | +Glucagon | | −Glucagon | |
| Test substance§ | (a) | (b) | (a) | (b) |
| Tween-80 | 25.8 ± 1.1 | (100%) | 22.2 ± 0.5 | (100%) |
| A - Oil fraction | 20.5 ± 1.6 | (80%) | 26.9 ± 2.3 | (100%) |
| B - Crude extract | 21.7 ± 2.1 | (84%) | 22.0 ± 1.1 | (99%) |
| C - Saponin-free extract | 20.6 ± 0.2* | (80%) | 24.9 ± 1.0 | (112%) |
| D - Saponin extract | 22.7 ± 3.6 | (88%) | 23.5 ± 1.0 | (106%) |
| E - Aglycone extract | 21.6 ± 0.7* | (84%) | 25.2 ± 1.9 | (113%) |
| Diosgenin | 20.4 ± 0.3* | (79%) | 22.5 ± 1.9 | (101%) |
| Trigonelline | 23.1 ± 1.2 | (89%) | 23.1 ± 0.8 | (104%) |

Data given as mean ± SEM of three separate hepatocyte preparations (n = 3 for each preparation). §Fractions were tested at 3.3 mg/ml in experiments without glucagon and 1.65 mg/ml with glucagon. (a) Results expressed as nmol glucose/mg protein/min, (b) results expressed as a % of the Tween-80 control. * = $p < 0.05$, with respect to Tween-80 (control).

### RESULTS AND DISCUSSION

Figure 1 shows typical profiles of glucose uptake into BBMV obtained in control (0.3% Tween 80) incubations and with 2 fenugreek fractions. All extracts as well as diosgenin and trigonelline were found to decrease the area under the glucose uptake profile without altering the equilibrium glucose concentration at 3600 s, indicating that the integrity of the BBMV was maintained. Tween 80 (0.3% v/v), was also found not to alter the integrity of the vesicles compared to water as shown by a non-significant difference in peak glucose uptake (Tab. I) and no difference in the equilibrium value at 3600 s (data not shown). Furthermore, we have found[32] that the addition of phloridzin (an SGLT-1 inhibitor[26]) decreases peak glucose uptake in a dose-dependent manner ($IC_{50}$ of 250 μM[32]), thus validating the use of this model for the study of glucose uptake *via* SGLT-1. Although GLUT-2 transporters are primarily located in the basolateral membrane of the enterocytes, their glucose-induced recruitment to the brush border membrane has clearly been demonstrated in perfused rat jejunum.[33] Indeed lower levels of GLUT-2 could be detected in rat BBMV even in the absence of glucose in the perfusate.[33] Nevertheless, it seems unlikely that GLUT-2 mediated transport occurs to any significant extent in the BBMV model reported in the present study since the uptake of labelled glucose into the vesicles was unaffected by the presence of up to 8 mM fructose (data not presented), whereas fructose and glucose are known to exert mutual inhibition of uptake *via* GLUT-2 transporters.[34]

With the exception of the petroleum ether extract (oil fraction, A) all the extracts significantly reduced ($p < 0.0001$) glucose uptake at the higher or both concentrations (Tab. I). Both layers derived from partitioning of the crude ethanol extract (B) inhibited uptake, indicating that both saponin (D) and non-saponin (C) constituents may be involved. There was no loss of activity on hydrolysing the saponins, indeed the greatest inhibition of glucose uptake was seen in the hydrolysed extract (E), believed to contain sapogenins (aglycones). On further fractionation of (E) by flash chromatography the main fraction G from this extract inhibited glucose uptake by 46%; whereas the other subfractions showed weaker inhibition (data not shown). The observed inhibition of glucose uptake in the aglycone fraction and its subfraction did not appear to be due to diosgenin or trigonelline, since no diosgenin or trigonelline could be detected in these fractions by TLC. Furthermore, diosgenin and trigonelline at 3.3 mg/ml inhibited glucose uptake by only 45% and 55% as compared to the 81% inhibition by 3.3 mg/ml of Extract (E). Of these two compounds diosgenin (8 mM) was more potent than trigonelline (19.2 mM). This suggests that diosgenin and trigonelline are not solely responsible for the effects seen with the crude extract. Further, in comparison to phloridzin ($IC_{50}$ 0.3 mM)[32] these two compounds exhibited very weak inhibition of this transporter.

Analysis of the sugar content of the extracts showed the presence of small quantities of glucose (0.016 mM at 3.3 mg/ml) and a negligible amount of fructose in the crude extract (B), considerably less in other extracts (< 0.01 mM) and none in the hydrolysed extract (E). This confirms that the inhibition of radiolabelled glucose uptake into BBMV by the fractions was not due to competition by unlabelled glucose in the fractions at the concentrations used.

The effect of the fenugreek extracts on the activity of HGPa activity is shown in Table II. None of the extracts (at 3.3 mg/ml) inhibited the enzyme in the absence of glucagon, suggesting that an insulinomimetic effect was not present. By contrast, in the presence of glucagon, fenugreek extracts (at 1.65 mg/ml) inhibited the enhanced HPGa activity by between 11–20% as compared to the control. However, this was only statistically significant ($p < 0.05$) with the saponin-free C (18.9%) and the sapogenin E (15.1%) fractions and diosgenin. The observed inhibition of HGPa activity in the aglycone fraction is unlikely to be due to diosgenin or trigonelline, since these compounds could not be detected in this fraction by silica-gel TLC. Nevertheless, diosgenin, but not trigonelline, affected HGPa activity. At 8 mM diosgenin significantly inhibited the enzyme activity by 20%. These results suggest that some components of fenugreek may be able to counteract the effects of the hormone glucagon. Fenugreek methanol and water extracts have also been shown to reduce HGPa activity induced by adrenaline in hepatocytes.[35]

In conclusion, these results show that fenugreek seeds contain substances capable of inhibiting the SGLT-1 mediated absorption of glucose across the brush border membrane of intestinal vesicles. The most active substance(s) with regard to inhibition of SGLT-1 seem to be non-lipid in nature, with the aglycone extract showing greatest activity. The results may well be relevant to the *in vivo* situation in that concentrations of about 3 mg/mL or less of fenugreek extract are attainable in the intestine, and the moieties reaching the small intestine are more likely to be aglycones than saponin glycosides (which would be hydrolysed by stomach acids). The active compound(s) are unlikely to be, but may be related to, diosgenin or trigonelline, two compounds previously associated with the medicinal properties of fenugreek. Further work to characterise the mechanism (competitive or non-competitive) by which SGLT-1 is inhibited by these extracts could only be undertaken once pure compounds are isolated from the active fractions, as the use of mixtures may complicate the results of such experiments.

Fenugreek seeds also seem to contain substances capable of affecting hepatic glycogen metabolism by inhibiting glucagon-induced HGPa activity in isolated liver hepatocytes. This activity was associated with the aglycone and saponin-free extracts. Although only a weak effect was observed at a concentration of 1.65 mg/mL, it is possible that the crude extracts used contain low concentrations of the actual active species, which if isolated would be effective at lower, and therefore more physiologically relevant concentrations.

These potential intestinal and hepatic effects of constituents of fenugreek seeds may explain the anti-diabetic effects of fenugreek observed by other researchers in various human and animal models.[4]

*Acknowledgement*

The authors thank the British Council (Yemen) for financing these investigations by the award of a grant to MAH.

### *References*

[1] Harris, M. I., Couric, C. C., Reiber, G., Boyko, E., Stern, M. and Bennet, P. (1995). *Diabetes in America*. 2nd edn. U.S. Printing office, NIH publication. Washington DC.

[2] Balkau, B., Shipley, M., Jarrett, R. J., Pyorala, K., Pyorala, M., Forhan, A. and Eschwege, E. (1998). High blood glucose concentration is a risk factor for mortality in middle-aged nondiabetic men, *Diabetes Care*, **21**, 360–367.

[3] Harris, M. I. and Eastman, R. C. (1998). Is there a glycaemic threshold for mortality risk?, *Diabetes Care*, **21**, 331–333.

[4] AL-Habori, M. and Raman, A. (1998). Anti-diabetic and hypocholesterolaemic effects of fenugreek, *Phytotherapy Res.*, **12**, 233–242.

[5] Sharma, R. D. (1986). Effect of fenugreek seeds and leaves on blood glucose and serum insulin responses in human subjects, *Nutr. Res.*, **6**, 1353–1364.

[6] Madar, Z., Abel, R., Samish, S. and Arad, J. (1988). Glucose lowering effect of fenugreek in Non-Insulin Dependent Diabetics, *Eur. J. Clin. Nutr.*, **42**, 51–54.

[7] Sharma, D., Raghuram, T. C. and Sadhukar Rao, N. (1990). Effect of fenugreek on blood glucose and serum lipids in Type I diabetes, *Eur. J. Clin. Nutr.*, **44**, 301–306.

[8] Valette, G., Sauvaire, Y., Baccou, J. C. and Ribes, G. (1984). Hypocholesterolaemic effect of fenugreek seeds in dogs, *Atherosclerosis*, **50**, 105–111.

[9] Ribes, G., Sauvaire, Y., Baccou, J. C., Valette, G., Chenon, D., Trimble, E. R. and Loubatieres-Mariani, M. M. (1984). Effects of fenugreek seeds on endocrine pancreatic secretions in dogs, *Ann. Nutr. Metab.*, **28**, 37–43.

[10] Ribes, G., Sauvaire, Y., Da Costa, C., Baccou, J. C. and Loubatieres-Mariani, M. M. (1986). Anti-diabetic effects of subfractions from fenugreek seeds in diabetic dogs, *Proc. Soc. Exp. Biol. Med.*, **182**, 159–166.

[11] Madar, Z. (1984). Fenugreek (*Trigonella foenum-graecum*) as a means of reducing postprandial glucose level in diabetic rats, *Nutr. Report. Int.*, **29**, 1267–1273.

[12] Amin, R., Abdul-Ghani, A. S. and Suleiman, M. S. (1987). Effect of *Trigonella foenum-graecum* on intestinal absorption, *Diabetes*, **36**(supp. 1), 211A.
[13] Platel, K. and Srinivasan, K. (1996). Influence of dietary spices or their active principles on digestive enzymes of small intestinal mucosa in rats, *Int. J. Food Sci. Nutr.*, **47**, 55–59.
[14] Moorthy, R., Parabhu, K. M. and Murthu, P. S. (1989). Studies on the isolation and effect of an orally active hypoglycaemic principle from the seeds of fenugreek (*Trigonella foenum-graecum*), *Diabetes Bull.*, **9**, 69–72.
[15] Ali, L., Azad Khan, A. K., Hassan, Z., Mosihuzzaman, M., Nahar, N., Nasreen, T., Nur-e-Alam, M. and Rokeya, B. (1995). Characterization of the hypoglycaemic effects of *Trigonella foenum-graecum* seed, *Planta Med.*, **61**, 358–360.
[16] Petit, P., Sauvaire, Y., Ponsin, G., Manteghetti, M., Fave, A. and Ribes, G. (1993). Effect of a fenugreek seed extract on feeding behaviour in the rat: Metabolic - Endocrine correlates, *Pharmacol. Biochem. Behav.*, **45**, 369–374.
[17] Hillaire-Buys, D., Petit, P., Manteghetti, M., Baissac, Y., Sauvaire, Y. and Ribes, G. (1993). A recently identified substance extracted from fenugreek seeds stimulates insulin secretion in rat, *Diabetologia*, **36**, A119.
[18] Sauvaire, Y., Baissac, Y., Leconte, O., Petit, P. and Ribes, G. (1996). Steroid saponins from fenugreek and some of their biological properties, *Adv. Exp. Med. Biol.*, **405**, 37–46.
[19] Sauvaire, Y., Petit, P., Broca, C., Manteghetti, M., Baissac, Y., Fernández-Alvarez, J., Gross, R., Roye, M., Leconte, A., Gomis, R. and Ribes, G. (1998). 4-Hydroxyisoleucine: A novel amino acid potentiator of insulin secretion, *Diabetes*, **47**, 206–210.
[20] Schmitz, J., Preiser, H., Maestracci, D., Ghosh, B. K., Cerda, J. J. and Crane, R. K. (1973). Purification of the human intestinal brush border membrane, *Biochimica et Biophysica Acta.*, **323**, 98–112.
[21] Kessler, M., Acuto, O., Storelli, C., Murer, H., Muller, M. and Semenza, G. (1978). A modified procedure for the rapid preparation of efficiently transporting vesicles from small intestinal brush border membranes: Their use in investigating some properties of D-glucose and choline transport systems, *Biochim. Biophys. Acta.*, **506**, 136–154.
[22] Fedorak, R. N., Chang, E. B., Madara, J. L. and Field, M. (1987). Intestinal adaptation to diabetes. Altered $Na^+$-dependent nutrient absorption in streptozotocin-treated chronically diabetic rats, *J. Clin. Invest.*, **79**, 1571–1578.
[23] Fedorak, R. N., Cheeseman, C. I., Thomson, B. R. and Porter, V. M. (1991). Altered glucose carrier expression: mechanism of intestinal adaptation during streptozotocin-induced diabetic rats, *Am. J. Physiol.*, **261** (Gastrointest. Liver. Physiol. 24), G585–G591.
[24] Burant, C. F., Flink, S., Depaoli, M., Chen, J., Lee, W. S., Hediger, M. A., Buse, J. B. and Chang, E. B. (1994). Small intestine hexose transport in experimental diabetes. Increased transporter mRNA and protein expression in enterocytes. *J. Clin. Invest.*, **93**, 578–585.
[25] Maenz, D. D. and Cheeseman, C. I. (1987). The $Na^+$-independent D-glucose transporter in the enterocyte basolateral membrane: orientation and cytochalasin B binding characteristics, *J. Membrane Biol.*, **97**, 259–266.
[26] Ferguson, D. R., Matthews, E. K., O'Connor, M. D. L. and Schuz, A. D. (1981). The interaction of phenformin and phlorizin with brush border membrane vesicles, phospholipid liposomes and phospholipid liposomes containing brush border membrane protein, *Biochem. Pharmacol.*, **30**, 1613–1619.
[27] Thorens, B. (1996). Glucose transporters in the regulation of intestinal, renal and liver glucose fluxes (Review), *Am. J. Physiol.*, **270** (Gastrointest. Liver. Physiol. 33), G541–G553.
[28] Al-Shibani, N. and Skett, P. (1994). Effect of guanamines on glycogen phosphorylase a activity in cultured hepatocytes from streptozotocin-diabetic rats, *Brit. J. Pharmacol.*, **113**, P 97.
[29] Bradford, M. M. (1976). A rapid and sensitive method for the quantitation of microgram quantities of protein utilizing the principle of protein-dye binding, *Anal. Biochem.*, **72**, 248–254.
[30] Hopfer, U., Nelson, K., Perrotto, J. and Isselbacher, K. J. (1973). Glucose transport in isolated brush border membrane from rat small intestine, *J. Biol. Chem.*, **248**, 25–32.
[31] Seglen, P. O. (1976). Preparation of isolated rat liver cells, *Method. Cell. Biol.*, **13**, 29–83.
[32] Vedavanum, K., Srijayanta, S., O'Reilly, J., Raman, A. and Wiseman H. (1999). Antioxidant action and potential antidiabetic properties of an isoflavonoid-containing Soybean phytochemical extract (SPE), *Phytother. Res.*, **13**, 601-608.
[33] Kellet, G. L. and Helliwell, P. A. (2000). The diffusive component of intestinal glucose absorption is mediated by the glucose-induced recruitment of GLUT2 to the brush-border membrane, *Biochem. J.*, **350**, 155–162.
[34] Garrriga, C., Moreto, M. and Planas, J. M. (1997). Hexose transport across the basolateral membrane of the chicken jejunum, *Am. J. Physiol.*, **272** (Regulatory Integrative Comp. Physiol. 41), R1330–R1335.
[35] Raman, A. and Skett, P. (1998). Traditional remedies and diabetes treatment. In: *Plants for Food and Medicine*, edited by Prendergast, H. D. V., Etkin, N. L., Harris, D. R. and Houghton, P. J., pp. 361–372. London, Royal Botanic Gardens, Kew.

